# Integrative Taxonomy of the Gall Mite *Nothopoda todeica* n. sp. (Eriophyidae) from the Disjunct Afro-Australasian Fern *Todea barbara*: Morphology, Phylogeny, and Mitogenomics †

**DOI:** 10.3390/insects14060507

**Published:** 2023-05-31

**Authors:** Philipp E. Chetverikov, Charnie Craemer, Vladimir D. Gankevich, Anna S. Zhuk

**Affiliations:** 1 Zoological Institute of Russian Academy of Sciences, Universitetskaya Nab. 1, 199034 St. Petersburg, Russia; vd.gankevich@gmail.com; 2 Manaaki Whenua–Landcare Research, 231 Morrin Road, Auckland 1072, New Zealand; charniecc@gmail.com; 3 Institute of Applied Computer Science, ITMO University, 191002 St. Petersburg, Russia; ania.zhuk@gmail.com

**Keywords:** eriophyoid mites, phytophagy, fern, disjunct distribution, mitochondrial genome

## Abstract

**Simple Summary:**

Plant-feeding gall mites of the superfamily Eriophyoidea are serious pests in agriculture because they are capable of transmitting viruses and causing growth abnormalities in plants. Evolutionary archaic gall mites are associated with ancient conifer hosts, while more derived forms inhabit flowering plants. In this study, we investigate the morphology and phylogeny of a new species of the subfamily Nothopodinae, *Nothopoda todeica* **n. sp.**, collected in South Africa from a fern, *Todea barbara.* This fern has an ancient distribution in Africa and Australasia and belongs to the Gondwanan royal fern family Osmundaceae. We show that the new species is not in a basal position in Nothopodinae. It is very similar and closely related to other members of *Nothopoda*, associated with derived groups of Asian flowering plants. This contradicts the expectation that the ancient host plant (fern) should be associated with a primitive mite. We also obtained a complete sequence of the mitochondrial DNA of *N. todeica* **n. sp.** And demonstrated that it has the same, but differently ordered, mitochondrial genes that were previously found in other eriophyoid mites. Our study contributes to the problem of the history of symbiotic relations of eriophyoid mites with plants, and provides new data that are important for better understanding their evolution.

**Abstract:**

Eriophyoidea is a group of phytoparasitic mites with poorly resolved phylogeny. Previous studies inferred Eriophyidae s.l. as the largest molecular clade of Eriophyoidea, and Nothopodinae as the basal divergence of Eriophyidae s.l. We investigate the morphology and molecular phylogeny of *Nothopoda todeica* **n. sp.** (Nothopodinae, Nothopodini), associated with a disjunct Afro-Australasian fern *Todea barbara* (Osmundaceae) from South Africa. Our analyses (1) determine new erroneous sequences (KF782375, KF782475, KF782586) wrongly assigned to Nothopodinae instead of Phyllocoptinae, (2) confirm the basal position of Nothopodinae in Eriophyoidea s.l., (3) question the monophyly of the Colopodacini and Nothopodini tribes, and (4) show the nested position of African fern-associated *Nothopoda* within a clade dominated by Asian nothopodines from angiosperms, which implies (a) a secondary association of nothopodines with ferns and (b) no relation between geography (continents) and the phylogenetic relationships of Nothopodinae species. Finally, we obtained a first complete mitochondrial genome for Nothopodinae and revealed a new gene order in the mitogenome of *N. todeica* **n. sp.**, notably deviating from those in other investigated eriophyoids. Our results contribute to resolving the phylogeny of Eriophyoidea and provide an example of an integrative study of a new taxon belonging to an economically important group of acariform mites.

## 1. Introduction

Eriophyoidea is an ancient group of greatly miniaturized and morphologically simplified acariform mites associated with higher vascular plants [1,2,3]. For decades, they were considered members of the cohort Eupodina within the suborder Trombidiformes [4]; however, recent comprehensive, morphological, multigene, and phylogenomic studies indicate their closest relation to a group of basal acariform soil mites of the family Nematalycidae outside Trombidiformes [5,6,7,8,9]. The phylogeny of Eriophyoidea is still poorly understood. Several studies have attempted to resolve the phylogeny of Eriophyoidea based on the analyses of fragments of mitochondrial (*Cox1*, *12S*, *16S*) and nuclear (*18S*, *28S*) genes [10,11,12,13]. All of them produced partially resolved trees with numerous clades conflicting with contemporary morphological systematics of this taxon [14]. Moreover, some of these studies were performed using suboptimal methodologies (e.g., with the inclusion of misidentified species, contaminated DNA templates, chimeric and/or inadequately aligned sequences), which call into question the conclusions of these works [15,16]. 

**Figure 1:**
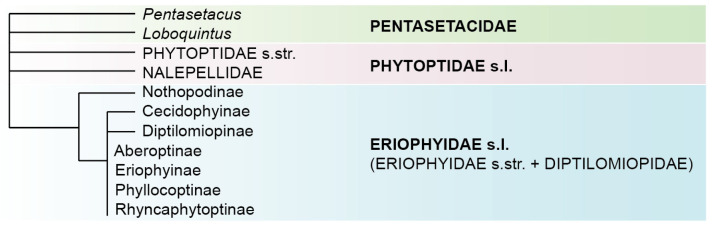
A cladogram summarizing the main hypotheses for the basal divergence of Eriophyoidea from molecular phylogenetic studies reported in the last decade. Putative large lineages of Eriophyoidea are in bold and given according to [11,18].

The consensus, at present, among all the published molecular cladograms of Eriophyoidea is a polytomy (Figure 1) comprising three large monophyletic lineages (Nalepellidae, Phytoptidae s.str., and Eriophyidae s.l.). The position of two smaller clades (*Pentasetacus* and *Loboquintus*) associated with ancient conifer hosts (*Araucaria* and *Cupressus*) and tentatively combined in the family Pentasetacidae [17,18,19] remains unresolved. They might represent basal eriophyoids or may be nested within the above-mentioned three large clades. Eriophyidae s.l. is the largest molecular clade of Eriophyoidea comprising various taxa of the morphologically defined families of Eriophyidae and Diptilomiopidae [14], which are non-monophyletic in all published molecular phylogenetic analyses. Within Eriophyidae s.l., three subfamilies (Nothopodinae, Cecidophyinae, and Diptilomiopinae) are often recovered to be monophyletic, with Nothopodinae usually representing the basal clade, whereas all other subfamilies (Eriophyinae, Phyllocoptinae, Aberoptinae, and Rhyncaphytoptinae) are inferred para- or polyphyletic [10,12,13]. Therefore, the basal divergence of Eriophyoidea is still unresolved, and the phylogenetic relations between the lower taxa (most tribes and genera) are unclear. 

This deadlock might be broken using a phylogenomic approach through the analyses of larger sets of gene sequences from full nuclear and mitochondrial genomes of Eriophyoidea. To date, there exist only one transcriptome (*Fragariocoptes setiger*), two full genomes (*Aculops lycopersici*, *F. setiger*), and six mitochondrial genomes (*A. lycopersici*, *Epitrimerus sabinae*, *F. setiger*, *Leipothrix* sp., *Phyllocoptes taishanensis*, and *Rhyncaphytoptus shagoanense*) of eriophyoid mites in GenBank (accessed on 1 May 2023) [8,12,20,21,22]. Genomic and transcriptomic data have recently been included in an analysis focusing on the phylogenetic position of Eriophyoidea in Acariformes that recovered eriophyoids as the sister group to nematalycids [8]. All published mitogenomes of eriophyoids share the same order of protein-coding genes, but differ in the position of the control region, rRNA, and several tRNA genes [8,12,20,22]. The differences in the gene order in mitogenomes may be promising molecular characters useful for testing phylogenetic relations within Eriophyoidea; therefore, the increase in the number of annotated mitogenomes of Eriophyoidea is needed for intensifying this line of research.

**Figure 2:**
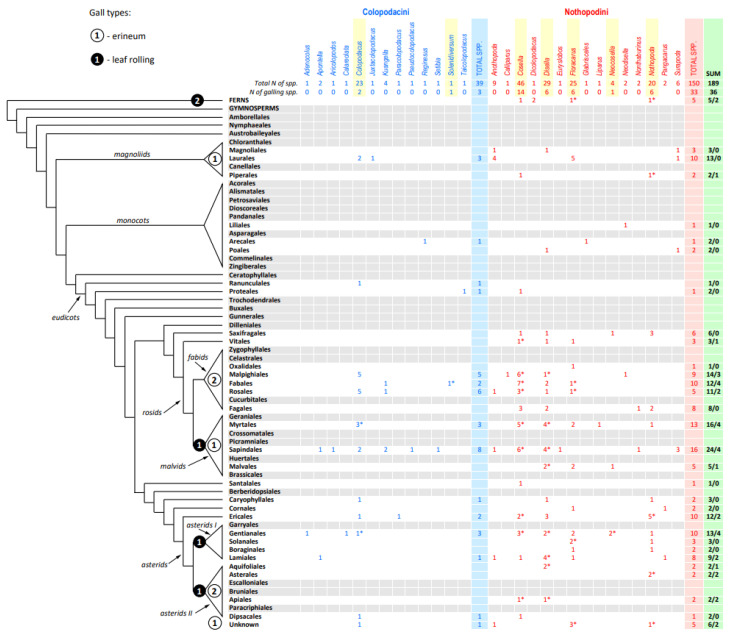
Distribution of Colopodacini (blue) and Nothopodini (red) mite species (right) on plant phylogeny (left). The plant phylogeny follows [23] with simplifications. Total numbers of species and numbers of species causing any type of damage (including leaf discoloration, deformation, marginal leaf rolling, and erineum) are given under the corresponding mite genus (highlighted in yellow). Asterisks indicate presence of galling species and species causing discoloration. The last column (green) shows total numbers of Nothopodinae species associated with plant orders/causing any galls on plants of this order. The numbers of nothopodine species causing two gall types (erineum and marginal leaf rolling) that are accompanied by high plant cell proliferation are shown on plant phylogeny within black (leaf rolling) and white (erineum) circles. Gray background indicate plant orders not inhabited by nothopodines.

Subfamily Nothopodinae Keifer is a morphologically distinct lineage of eriophyids characterized by their synapomorphic modification of legs: their tibiae are greatly reduced or completely absent [14]. According to our estimates, based on a comprehensive literature search, this subfamily comprises 189 species divided into two tribes differing in the presence (Colopodacini, 39 species, 13 genera) or absence (Nothopodini, 150 species, 15 genera) of coxal setae *1b*. Nothopodinae are absent from gymnosperms and early angiosperms (Figure 2). A few species are associated with early-derivative plant clades (ferns—5 spp., magnoliids—18 spp., and monocots—5 spp.), most of which (24 of 28) belong to the tribe Nothopodini. The majority of Nothopodinae are associated with eudicots, with the highest species numbers for malvids, fabids, and asterids I. Among all plant orders, Sapindales is the most highly inhabited by Nothopodinae (24 spp.), followed by Laurales, Malpighiales, Rosales, Mirtales, Ericales, and Gentianales (11–16 spp. each). Remarkably, the three plant orders most inhabited by Colopodacini and Nothopodini (Sapindales, Malpighiales, Rosales) coincide, indicating their possible long-term coexistence on shared host plant lineages.

Following the distribution of their host plants, nothopodines are predominantly distributed in a temperate-climate zone with about 77% species described as being from Asia (mostly from China). In the boreal zones of Eurasia and North America, nothopodines have not been recorded; although, several species (including introduced ones) are known to be from southern Europe and USA. In Oceania, South America, and Africa, Nothopodinae are limited and comprise, in total, about 25 species.

Similar to the entire superfamily Eriophyoidea [24], most species of Nothopodinae are vagrant, causing no apparent damage to their hosts. Only 39 species from seven genera (*Colopodacus*, *Solenidiversum*, *Cosella*, *Disella*, *Floracarus*, *Nothopoda*, and *Nonthaburinus*) cause various galls and discolorations on plants; most of them (~92%) belong to the tribe Nothopodini. No Nothopodinae species induce the formation of galls containing a true chamber sensu [25], similar to pouch or nail galls [26]. Most damage is related to leaf discoloration, deformation, and curling. The only two types of galls that are induced by Nothopodinae and accompanied by intensive plant cell growth and proliferations are erinea and marginal leaf rolling. They are rare and do not group together when labeled in plant phylogeny, but are scattered in different plant clades (Figure 2).

In 2013–2017, several expeditions focusing on exploring the diversity of eriophyoid mites associated with indigenous Southern African plants were organized by the ARC Plant Protection Research Institute (Roodeplaat, Gauteng, South Africa). During these expeditions, numerous samples containing eriophyoids were collected from four provinces in South Africa (Gauteng, Mpumalanga, Limpopo, and Western Cape). Among them, only one sample that was collected from an indigenous fern, *Todea barbara* (L.) T. Moore in the Western Cape, contained nothopodine mites. *Todea barbara* has a disjunct Afro-Australasian distribution and belongs to the Triassic leptopteroid clade of the ancient late-Paleozoic fern lineage Osmundaceae [27,28]. This lineage experienced mass extinction. It has a uniquely rich and diverse fossil record, and the extant osmundaceans, including *T. barbara*, are considered “living fossils” [29,30].

In this paper, we describe a new eriophyoid mite species of the genus *Nothopoda* from *T. barbara*, provide the sequences of *Cox1* and rDNA genes, describe the complete mitogenome, and compare it to other published mitogenomes of Eriophyoidea. We also reconstruct a molecular phylogeny of Nothopodinae using the data available in GenBank in order to test three hypotheses: (1) species of Nothopodinae cluster according to the presence/absence of coxal seta *1b* and form two clades corresponding to the Nothopodini and Colopodacini tribes, (2) the phylogenetic relationships of Nothopodinae species is related to the continent on which they occur, and (3) cladogenesis of Nothopodinae follows the divergence of higher vascular plants into large superclades, specifically–ferns and angiosperms.

## 2. Materials and Methods

**Collection and morphological measurements.** The fronds of the fern *Todea barbara* (L.) T. Moore were sampled in South Africa in November 2016. They were examined under a stereo microscope and the mites were collected using a minuten pin. Some mites were slide-mounted in modified Berlese medium with iodine [31] and cleared on a heating block at 90 °C for 3–5 h. The rest of the mites were stored in Eppendorf tubes filled with 96% ethanol and kept in a refrigerator (−25 °C) for DNA extraction. The external morphology of the slide-mounted specimens was studied using conventional light microscopy (LM) using a Leica DM2500 and photographed with a ToupCam E3ISPM05000KPA digital camera. Morphological descriptions were based on phase contrast (PC) and differential interference contrast (DIC) LM observations. All measurements were obtained using ToupTek ToupView software. They are presented in the descriptions in micrometers (µm) and present lengths, except when otherwise stated. The measurements of the females were based on the holotype, whereas the ranges (in brackets) were based on the measurements of para- and holotypes. In the descriptions of males, only ranges were presented. The terminology of eriophyoid morphology and classification of Eriophyoidea follow [4,14], respectively. Drawings of mites were sketched by pencil using a video projector [32], scanned, and finalized in Adobe Illustrator CC 2014 (Adobe Systems, San Jose, CA, USA) using a Wacom Intuos S CTL-4100K-N (Wacom Co., Ltd., Kazo, Saitama, Japan) graphics tablet.

**DNA extraction and sequencing.** For DNA extraction, four females from two different populations (mentioned below in the “Type Material” and “Additional Material” subections of the Section 3.1) were crushed separately with a fine pin in a 2 μL drop of distilled water on a cavity-well microscope slide. The fragments of the anterior part of the mite were pulled out of the drop and slide-mounted to verify the species identity. Each drop was pipetted into a thin-walled PCR tube with 20 μL of 12% solution of Chelex**^®^** 100 Resin (Bio-Rad Laboratories, Inc., Hercules, CA, USA) before being heated 3 times (5 min at 95 °C) in a thermostat with intermediate short vortexing. The solution above the Chelex^®^ granules was used as the DNA template for PCR to amplify the fragments of the *Cox1* gene. For the PCR and sequencing, we applied the protocol and primers detailed by [7]. *Cox1* sequences were obtained using BigDye Terminator v.3.1 chemistry (Applied Biosystems, Foster City, CA, USA) and a 3500xl Genetic Analyzer (Applied Biosystems). Sequences of *18S* and *28S* rDNA genes were obtained through genomic sequencing (see the subsection “Mitogenomics” in the end of Section 2 below).

**Sequence alignment and molecular phylogenetic analyses.** Molecular phylogenetic analyses of *18S, 28S*, and *Cox1* sequences of eriophyoid mites were performed to assess the phylogenetic positions of the new nothopodine species and its relationships with members of Nothopodinae. For this purpose, we included all nothopodines that are currently present in https://www.ncbi.nlm.nih.gov/nucleotide/ (accessed on 23 March 2023) and added three species from Eriophyidae s.l. (*Epitrimerus sabinae*, *Leipothrix* sp., *Phyllocoptes taishanensis*, and *Rhinotergum shaoguanense*) for which complete *Cox1*, *18S*, and *28S* sequences are available. We then removed all identical sequences of the same species. We also removed all sequences of *Floracarus perrepae* (Nothopodini) and *Nonthaburinus* (Colopodacini), because they were too short and crushed the analysis because of too few numbers of common sites. The sequences of one species of Pentasetacidae (*Loboquintus subsquamatus*) and five species of Phytoptidae s.l. (*Fragariocoptes setiger*, *Novophytoptus rostratae*, *Oziella atherodes*, *O. hirta*, *Trisetacus piceae*, and *T. pini*) were used, respectively, as distant and close out-groups in our analyses. We combined them with the previously mentioned sequences of Eriophyidae s.l. and obtained three final FASTA files that included 28/29/19 sequences of *18S*/*28S*/*Cox1* genes. All sequences, except those of the new species, were generated in previous studies focusing on the molecular phylogenetics of Eriophyoidea [8,10,12,20].

Sequences of *18S* and *28S* genes were aligned with the E-INS-i MAFFT algorithm [33] through the web-based program interface [34] using default settings and the alignments from [35] as references. Subsequently, the reference sequences and gap-only sites were removed, and poorly aligned positions and divergent regions in the automated MAFFT alignments were eliminated with Gblocks [36,37], with the most stringent settings (smaller final blocks, gap positions within the final blocks, less-strict flanking positions, and many contiguous non-conserved positions were not allowed) implemented in the Web-based interface [38]. Sequences of the *Cox1* gene were treated as codons. Maximum likelihood analyses were conducted in IQ-tree 2 [39]. For the gene evolution, the TIM2 + F + I + I + R3 model was selected for merged *18S* + *28S* datasets and the GY + F3X4 + I + G4 model was selected for the *Cox1* dataset using ModelFinder [40], as implemented in IQ-tree 2 based on the Bayesian Information Criterion. Branch support values were generated from the Ultrafast bootstrap approximation (UFBoot) with 10,000 bootstrap alignments, 10,000 maximum iterations, and a minimum correlation coefficient of 0.99. Values of a single branch test (SH-like approximate likelihood ratio test, SH-aLRT) with 10,000 replicates and Ultrafast bootstrap support (UFBS) were labeled on the maximum likelihood (ML) trees.

**Mitogenomics.** In order to obtain the complete mitochondrial genome of the new nothopodine species, we extracted DNA from 400 ethanol-preserved mite specimens using a LumiPure Genomic DNA from AnySample Kit (Lumiprobe, Germany) and prepared genomic libraries using a Nextera DNA Flex Library Prep Kit. Genomic sequencing (150 bp paired reads) was performed using Illumina Hiseq 6000 in Eurogen (Russia). FastQC was used to calculate and visualize the sequence-quality metrics of raw and filtered reads [41]. Raw reads were filtered by AfterQC (v0.9.7) [42] to remove adapters, overrepresented sequences, and low-quality nucleotides (Q < 20). De novo assembly of the filtered reads were performed using SPAdes v.3.15.4 with metaparameter [43]. Mitochondrial scaffolds from a metagenome assembly were determined among the assembled contigs via searching for fragments of a previously sequenced *Cox1* gene (see above) by lastz v.1.04.00 (https://github.com/lastz/lastz, accessed on 23 March 2022). Additionally, the de novo assembly of the mitochondrial genome was performed by GetOrganelle v.1.7.7.0 [44] and MitoZ v.2.4 [45]. All mitochondrial genome assemblies were aligned by MAFFT v.7 and manually checked to select better consensus results in Unipro UGENE v.46 [46]. Ribosomal RNA genes (5.8S, 18S, 28S) in the metagenome assembly were determined by Barrnap v.0.9 with a parameter for kingdom eukaryotes [47]. Scaffold with rRNA genes was extracted from the metagenome assembly by *bedtools getfasta v.2.29.2* [48] or *seqtk subseq* (https://github.com/lh3/seqtk, accessed on 23 March 2022). BLASTn v.2.13.0 [49] against the nucleotide database was used to identify ribosomal RNA genes related to eriophyoid mites from all scaffolds with rRNA genes founded in the metagenome assembly. A draft annotation of the mitochondrial genome was obtained using the MITOS Web server [50] and Arwen [51]. Then, the mitochondrial sequence of *Nothopoda todeica* **n. sp.** was manually annotated in Unipro UGENE v.46 [46] using the mitochondrial genomes of eriophyoids from GenBank (NC029208, NC029209, KX027361, KX027362, CM034476) as references. 

For the molecular phylogenetic analysis, mitochondrial rRNA (*12S* and *16S*) and protein genes (translated into amino acids) of eight eriophyoid taxa (including the new species and one phytoptid mitochondrion, CM034476, used as the out-group) were aligned using MAFFT and the resulting alignments were modified using Gblocks, as described above. Sequences of the *ATP8* gene were excluded because Gblocks failed to find reliable blocks in the alignment of this gene. Maximum likelihood analyses were conducted in IQ-tree 2 [39]. For gene evolution, the GTR + F + I + G4 model was selected for merged *12S* + *26S* datasets, mtZOA + F + G4 was selected for merged *ATP6 + COX2 + COX3 + CYTB + NAD1 + NAD2 + NAD3 + NAD4 + NAD4L + NAD5 + NAD6* datasets, and the mtART + I + G4 model was selected for the *Cox1* dataset using ModelFinder [40], as implemented in IQ-tree 2 based on the Bayesian Information Criterion. All other steps of the analysis were similar to those described in the previous section.

## 3. Results

### 3.1. Taxonomy

#### *Nothopoda todeica* **n. sp.** (Figure 3 and Figure 4)

**FEMALE (*n* = 8).** Body fusiform, slightly yellowish, 168 (156–196), 60 (59–64) wide at level of setae *c2*. **Prodorsal shield** subtriangular, 36 (32–38), 75 (72–85) wide, without frontal lobe, anterior margin of prodorsal shield distinct, broadly rounded. Prodorsal shield with distinct net-like pattern formed by longitudinal and transverse ridges. Median and admedian lines complete, entire, admedians converging in anterior part of shield. Submedian line I indistinct or fragmented in posterior half of prodorsal shield and situated between admedian line and tubercle of *sc*. There are four rows of cells. Anterior-most row includes 9–10 cells formed by thin lines; these cells are open anteriorly, ending almost on the anterior margin of the shield. Second row includes six cells; among them two median cells are subrectangular and the remaining four cells are subtriangular or of a more irregular shape. The two posterior rows have one uniform rectangular cell on each side of the median line. Posterolateral areas of the prodorsal shield with distinct curved ridges that are dorsal continuations of ventral opisthosomal semiannuli. Tubercles of setae sc. large, situated anterior to prodorsal shield rear margin, sc. 15 (15–17), 25 (22–26) apart, directed upward and convergently posteriad. **Gnathosoma** projecting downward, palps 14 (12–15). Gnathosomal setae: seta *ν* 1 (1–2); pedipalp genual seta *d* absent in all studied specimens (*n* = 8); and pedipalp coxal seta *ep* 2 (1–3). Suboral plate is smooth and 6 (6–7), 8 (8–10) wide.

**Leg I** 23 (22–24), tarsus 7 (7–8), *u′* 3 (2–4)*, ft′* 22 (20–26), *ft”* 17 (15–21), *ω* 5 (5–6) with large spherical knob; empodium 4 (4–5), 4-rayed, all rays except terminal pair with one (in basal ray) or two (in other rays) additional subrays; tibia absent; genu 5 (4–5), *l”* 23 (21–26), femur 8 (7–9), *bv* 5 (5–6), with ventro-distal microgranulations. **Leg II** 21 (21–24), tarsus 7 (7–8), *u′* 3 (2–3)*, ft′* 6 (6–8), *ft”* 18 (16–21), *ω* 7 (6–7) with large spherical knob; empodium 4 (4–5), 4-rayed, similar to empodium I; tibia absent; genu 4 (4–5), *l”* 10 (8–11), femur 8 (8–9), *bv* 7 (6–8), with ventro-distal microgranulations. **Coxal plates I and II** with distinct spine-shaped microtubercles situated mainly around tubercles of setae *1a* and *2a*; coxal setae *1b* absent; *1a* 9 (8–9), 10 (9–11) apart; *2a* 22 (20–25), 22 (21–24) apart. Prosternal apodeme indistinct; 4 (3–5) incomplete coxigenital annuli between coxae II and epigynium. 

**External genitalia.** Genital coverflap posteriorly rounded and slightly notched midway, with two curved, fragmented transverse ridges in two rows separating thin, smooth, distal part of coverflap and thicker microtuberculated basal part, 10 (9–10), 20 (19–23) wide; setae *3a* 7 (6–7), 17 (16–19) apart. **Internal genitalia (*n* = 3).** Spermathecae spherical, 3–4 wide; very short spermathecal tubes, about 1, 1.5–2 wide, with narrow acuminate spermathecal process about 2 long; longitudinal bridge 5–7; anterior genital apodeme trapezoidal, bell-shaped, with fine microgranulations, oblique apodeme distinct, and sinuous. 

**Opisthosoma** dorsally with 62 (61–69) annuli, ventrally with 60 (59–64) annuli between posterior margin of coxae II and caudal lobes. Dorsal annuli smooth, except posterior-most 5–10 annuli with small, irregular, round microtubercles. Ventral annuli with spine-shaped microtubercles in anterior half of opisthosoma and more elongated, gradually changing their shape and becoming more elongated and eventually ridge-like, posterior to tubercles of setae *f*. Setal lengths: *c2* 23 (22–31), *d* 46 (42–48), *e* 6 (6–7), *f* 14 (14–15); *h1* 4 (3–5); *h2* 38 (37–45); 9 (9–10) annuli from rear shield margin to *c2*; 11 (10–12) annuli between *c2*–*d*; 13 (13–15) annuli between *d* and *e*; 18 (18–19) annuli between *e* and *f*; and 8 (8–9) annuli between *f* and *h2*. Small subrhomboid plate about 3–4, 3–4 wide present behind last ventral opisthosomal annulus. This plate is dotted. The dots may be small, round microtubercles or pores. Indistinct cuticular tubes and small rectal sac (the elements of the anal secretory apparatus, *ASA*, sensu [52]) were observed in one female.

**Type material.** Holotype female from slide E4719 and paratype females in slide series E4251, E4320, and E4322, and in vials filled with 96% ethanol collected on 7 November 2016 by P. Chetverikov, C. Craemer, and S. Neser on fronds of *Todea barbara* (L.) T. Moore in Cederberg Wilderness Area, near the Algeria Waterfall (32°21′41.8″ S 19°04′19.4″ E), Western Cape Province, South Africa (Figure 5). Type material is deposited in the Acarological Collection of the Zoological Institute of the Russian Academy of Science (ZIN RAS) in Saint-Petersburg (Russia) and the ARC Plant Protection Research Institute in Roodeplaat, Pretoria (South Africa).

**Host and relation to host.** Mites live on the lower surface of fronds of *Todea barbara* (L.) T. Moore (Osmundaceae), causing no visible damage.

**Remarks.** Paleobiogeography of *Todea*, the host genus of *N. todeica* **n. sp.**, suggests a largely disjunctive Gondwanan distribution of this fern genus across the Southern Hemisphere in the past with different species that were common in South America, Africa, and Australasia in the Cenozoic [29]. At present, *Todea* is absent in South America and comprises only two extant species: *T. papuana* and *T. barbara.* The first species is a New Guinea endemic, whereas *T. barbara* has a remarkable Afro-Australasian distribution with two relictual populations, one distributed in Southern Africa (Mozambique, South Africa, Swaziland, and Zimbabwe) and the other in Oceania (Australia and New Zealand) [28]. Our finding of *N. todeica* **n. sp.** on *T. barbara* in South Africa presents interesting questions for the future research: (1) does *Nothopoda* occur on *Todea* in Australasia and (2) which extant eriophyoid taxa are primary and which are secondary (e.g., shifted from angiosperms or gymnosperms) symbionts of ferns?

**Additional material.** Females and males from slide series E4721–E4723, F184, F186, and F187 were collected from the same host, by the same collectors on right bank of the Lower Palmiet River, in the Kogelberg Nature Reserve, Western Cape Province, South Africa (34°19′42.8″ S 18°58′50.5″ E) (Figure 5).

**GenBank data.** OQ737112 (*18S*, isolate type, 2300 bp), OQ737113 (complete *ITS1-5.8S-ITS2*, isolate type, 1250 bp), OQ737114 (*28S*, isolate type, 3252 bp), OQ720953 (partial *Cox1*, isolate type, 1223 bp), OQ720954 (partial *Cox1*, isolate additional, 1223 bp), and OQ934080 (*complete mitochondrion*, isolate type, 14018 bp).

**Figure 3:**
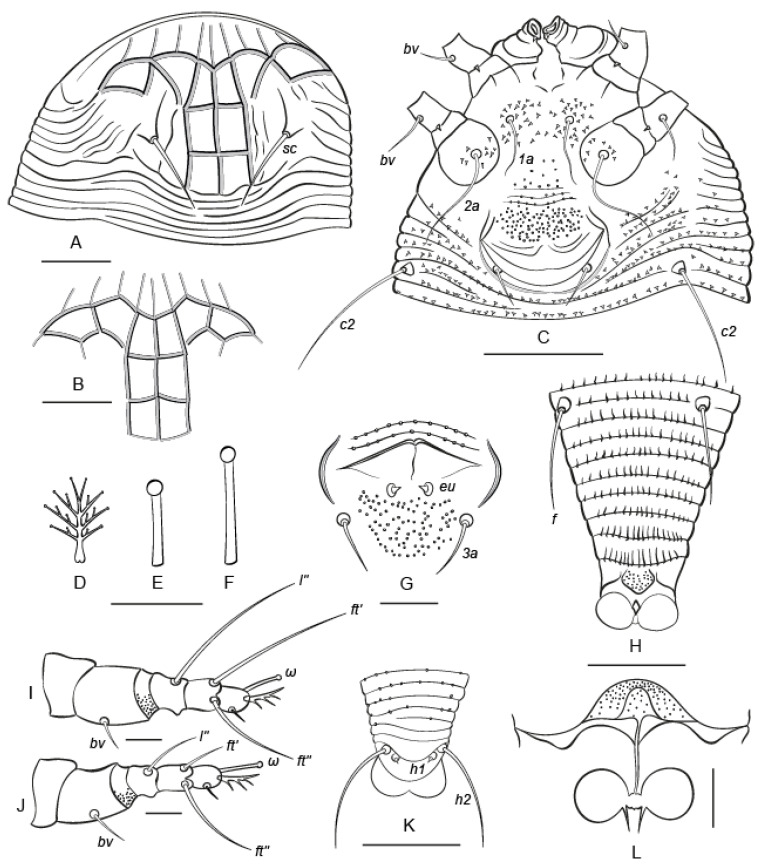
Drawings of *Nothopoda todeica* **n. sp.** (female). (**A**,**B**) Ornamentation of prodorsal shield, (**C**) coxigenital area, (**D**) empodium I, (**E**,**F**) tarsal solenidia I (**E**) and II (**F**), (**G**) male genital area, (**H**) ventral view of telosome, (**I**,**J**) legs I (**I**) and II (**J**), (**K**) dorsal view of caudal part of opisthosoma, (**L**) female internal genitalia. Scale bar: (**A**,**B**,**H**,**K**)—15 µm; (**D**–**G**,**I**,**J**,**L**)—5 µm; (**C**)—20 µm.

**Remarks.** Females of *N. todeica* **n. sp.** from the type (*n* = 8) and additional (*n* = 9) material morphologically were very similar, except that the females from the type locality were smaller (length of body 156–196 vs. 186–234), had narrower and shorter prodorsal shields (33–36 × 45–52 vs. 37–40 × 60–63), and differed slightly in the shape of the cells in the second row on the prodorsal shield (Figure 3A vs. Figure 3B). Males were found in the additional material only.

**MALE (additional material, *n* = 3).** Body fusiform, slightly yellowish, 171–200, 55–64 wide at level of setae *c2*. **Prodorsal shield** 35–40, 50–55 wide, without frontal lobe, anterior margin of prodorsal shield distinct and broadly rounded. Ornamentation of prodorsal shield similar to that in females. Setae *sc*. 14–15, 25–29 apart, directed upward and convergently posteriad. **Gnathosoma** projecting downward; palps 11–15. Gnathosomal setae: seta *ν* 2–3; pedipalp genual seta *d* absent; pedipalp coxal seta *ep* 2 (2–3). **Leg I** 21–23, tarsus 7–8, *u′* 2–3, *ft′* 19–24, *ft″* 18–20, *ω* 6–8 with large spherical knob; empodium 4–5, 4-rayed, similar to that in females; tibia absent; genu 4–5, *l″* 20–26, femur 7–8, *bv* 4–5. **Leg II** 19–21, tarsus 6–8, *u′* 2–3, *ft′* 6–7, *ft″* 17–19, *ω* 6–8 with large spherical knob; empodium 4 (4–5), 4-rayed, similar to empodium I; tibia absent; genu 4–5, *l″* 8–11, femur 7–9, *bv* 5–7. **Coxal plates I and II** with distinct spine-shaped microtubercles situated mainly around tubercles of setae *1a* and *2a*; coxal setae *1b* absent; *1a* 7–16, 10–11 apart; *2a* 14–17, 20–22 apart. Prosternal apodeme indistinct, 7–8 coxigenital annuli between coxae II and external genitalia. Genital area 15–18 wide, setae *eu* 0.5–1, *3a* 6–7, 13–14 apart, cuticle between tubercles of *3a* with small, round microtubercles. **Opisthosoma** dorsally with 58–65 annuli and ventrally with 57–66 annuli between posterior margin of coxae II and caudal lobes. Opisthosomal annuli similarly microtuberculated to those in females. Setal lengths: *c2* 18–25, *d* 34–39, *e* 4–6, *f* 13–15; *h1* 3–4; *h2* 34–42; 12–13 annuli from rear shield margin to *c2*; 8–13 annuli between *c2*–*d*; 12–13 annuli between *d* and *e*; 17–18 annuli between *e* and *f*; and 7–8 annuli between *f* and *h2*. 

**Etymology.** The species name, *todeica*, is a feminine adjective o in the nominative case. It is derived from the generic name of the host plant and means “associated with *Todea*”.

**Differential diagnosis.** Among all presently known species of the genus *Nothopoda*, the new species is morphologically closest to *Nothopoda camelliae* [53]. The main differences between *N. todeica* **n. sp.** and *N. camelliae* are in (a) the shapes of microtubercles on coxal plates, (b) pattern of distribution of microtubercles on opisthosomal annuli, (c) presence/absence of microgranulations on femora I and II, and (d) presence/absence of short thin lines between the thicker ridges of the prodorsal shield that form cells (Table 1).

**Remarks.** We also compared the new species with *Nothopoda natalensis* Meyer & Ueckermann, 1997, the only member of the tribe Nothopodini reported in Africa, and *N. footei* (Keifer, 1969), the only other *Nothopoda* species associated with ferns. Both species can be separated from *N. todeica* **n. sp.** based on the ornamentation of the prodorsal shield and female genital coverflap, and the presence/absence of microtubercles on dorsal opithosomal annuli (Table 2). 

**Table 2:** Morphological differences between *Nothopoda todeica* **n. sp.**, *N. natalensis* Meyer & Ueckermann, 1997, and *N. footei* (Keifer, 1969).

Character	*N. todeica* n. sp.	*N. footei*	*N. natalensis*
Median line of prodorsal shield	Entire, simple	Forked anteriorly	Entire, simple
Microtubercles on lateral areas of prodorsal shield	Absent	Present	Present
Cells along anterior margin of prodorsal shield	9–10, along whole anterior anterolateral margin	2–4, only in antero-medial region	2–4, only in antero-medial region
Ornamentation of genital coverflap	Two transverse rows of striae	Two longitudinal striae	14 transverse striae
Host plant	*Todea barbara* (L.) T. Moore (Osmundaceae)	*Nephrolepis* sp. (Nephrolepidaceae)	*Aspilia natalensis* (Sond.) Wild (Asteraceae)
Relation to host	Vagrant on lower surface of fronds	in terminal galls on the frond branches	in erineum within dome-like gall
Reference	This paper	[54]	[55]

**Figure 4:**
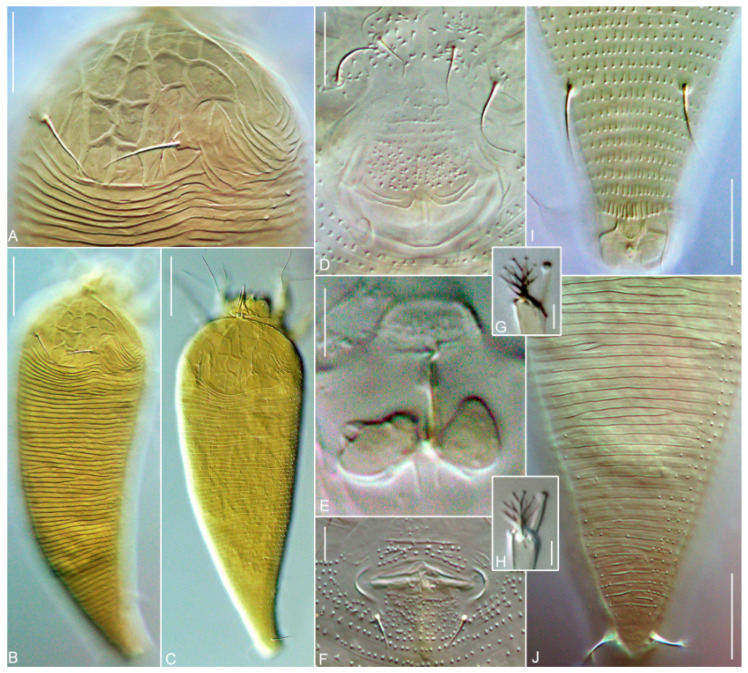
DIC microphotographs of *Nothopoda todeica* **n. sp.** (**A**) Female prodorsal shield, (**B**,**C**) dorsal views of female (**B**) and male (**C**), (**D**) female coxigenital area, (**E**) female internal genitalia, (**F**) male genital area, (**G**,**H**) empodium I and tarsal solenidion I of female (**G**) and male (**H**), (**I**,**J**) ventral (**I**) and dorsal (**J**) views of caudal part of opisthosoma. Scale bar: (**A**,**I**,**J**)—15 µm; (**B**,**C**)—30 µm; (**D**)—10 µm; (**E**,**F**)—5 µm; (**G**,**H**)—2 µm.

**Figure 5:**
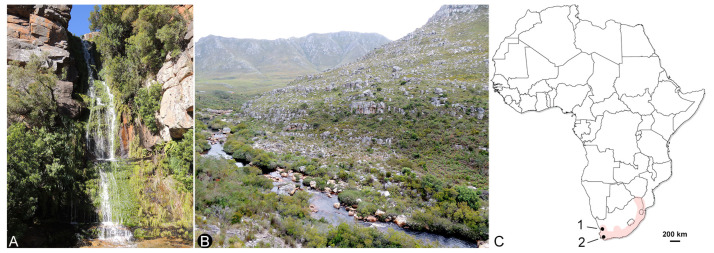
Collection sites of *Nothopoda todeica* **n. sp.** in Western Cape Province of South Africa. (**A**) Algeria Waterfall (site 1) in Cederberg Wilderness Area, the type locality of *N. todeica* **n. sp.** (**B**) canyon of Palmiet River in Kogelberg Nature Reserve (site 2), (**C**) estimated distribution of fern *Todea barbara* in Africa (colored pink, based on open data source from [56]) and collection sites 1 and 2.

### 3.2. Molecular Phylogenetics

#### 3.2.1. Blast Search Results for Cox1 and rDNA Sequence of *Nothopoda todeica* **n. sp.**

*Cox1* sequences of females of *N. todeica* **n. sp.** from the type (OQ720953) and additional (OQ720954) material were identical, except for one synonymous nucleotide substitution (A vs. R) in the 468 position of the *Cox1* gene in a codon corresponding to serin. 

*Cox1* sequences of *Nothopoda* spp. were absent from https://www.ncbi.nlm.nih.gov/nucleotide/ (accessed on 23 March 2023). A Blastn search for *Cox1* sequence OQ720953 of *N. todeica* **n. sp.** against Eriophyoidea returned as the best hits the sequences of two nothopodine species *Floracarus perrepae* (AY823158) and *Cosella viburniae* (MZ274943) when sorted by percentage of identity. Blastx returned sequences of *Cosella parvifoliae* (UOK10607, 51% coverage, 90.38% identity) and *C. viburniae* (QVU25152, 54% coverage, 82.19% identity) when filtered by coverage (>50%) and sorted by percentage of identity.

A Blast search for *18S* (OQ737112) and *28S* (OQ737114) sequences of *N. todeica* **n. sp.** against Eriophyoidea returned two sequences of *Nothopoda* sp., MZ279931 (*18S*, 96% coverage, 98.51% identity) and KF782476 (*28S*, 30% coverage, 93.54% identity) as the best hits, when filtered by E-value.

*ITS1-5.8S-ITS2* sequences of Nothopodinae were absent from GenBank (accessed on 23.03.2023). A Blast search for the *ITS1-5.8S-ITS2* sequence OQ737114 of *N. todeica* **n. sp.** against Eriophyoidea returned as the best-hits various sequences of Eriophyinae (*Aceria*, *Aculops*) and Cecidophyinae (*Cecidophyopsis*).

#### 3.2.2. Molecular Phylogenetic Analyses (Figure 6)

**Figure 6:**
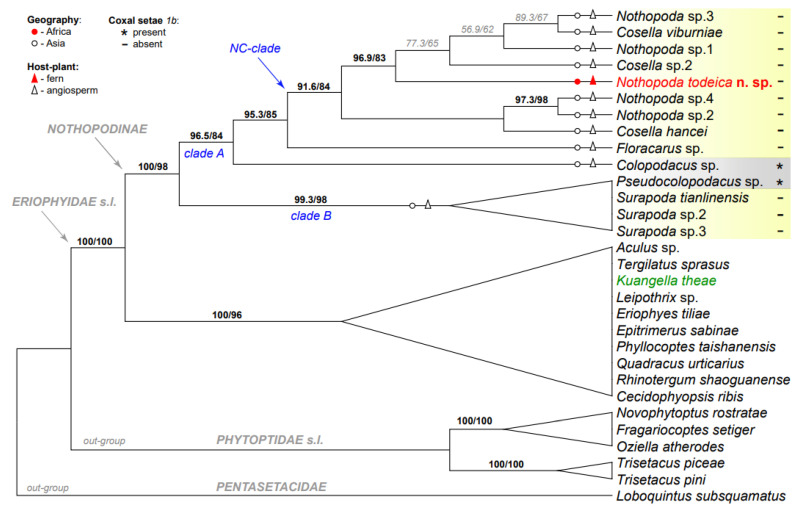
Maximum-likelihood phylogeny (*18S* + *28S* + *Cox1*) of Eriophyoidea showing phylogenetic position of *Nothopoda todeica* **n. sp.** in Nothopodinae. Branch labels are: SH-aLRT (%)/UFBS support (%); all values of UFBS > 80 and SH-aLRT > 90 are in bold. Three large nothopodine clades (A, B, NS) are indicated (blue). Mite species of the tribe Colopodacini are highlighted in gray and those of the tribe Nothopodini are highlighted in yellow. A single nothopodine taxon (*Kuangella theae*) that was inferred out of Nothopodinae is colored green. Labels for geography, host plants, and mite morphology are explained in the legend in the upper-left corner of the figure. Higher eriophyoid taxa are indicated with gray italic font.

Our analyses of *18S*, *28S*, and *Cox1* sequences produced a tree topology (Figure 6) showing the basal divergence of Eriophyoidea into the three main lineages (Pentasetacidae, (Phytoptidae s.l., Eriophyidae s.l.)). In all analyses, a colopodacine taxon *Kuangella theae* was constantly inferred as a member of a large clade of non-nothopodine taxa within Eriophyidae s.l. All other sequences of Nothopodinae formed a monophyletic group, dichotomously diverging into clades A and B, each including a series of moderately supported clades. The only two colopodacine taxa involved in our analyses (*Colopodacus* and *Pseudocolopodacus*) were inferred in different clades of Nothopodinae (in *A* and *B*, correspondingly) indicating (a) a putative homoplastic loss of coxal setae *1b* in Nothopodinae and (b) polyphyly of the tribes Colopodacini and Nothopodini. *Nothopoda todeica* **n. sp.**, a single African taxon from a fern host in our analyses, was nested within a large group of Asian nothopodine taxa associated with angiosperms in clade A. Sequences of two nothopodine genera, *Cosella* and *Nothopoda*, did not form separate genus-specific groups in our tree, but were mixed in a moderately supported clade *NC* that was sister to *Floracarus.*

**Remarks.** After inferring *Kuangella theae* in the unexpected phylogenetic position out of Nothopodinae, we performed a Blast search for the three sequences of this species (KF782375, KF782475, KF782586) obtained by [10], which we used in our analyses. All of them showed high similarities with the sequences of Phyllocoptinae: KF782475 (*K. theae*, isolate 28sd2-5r7 28S)—about 85% similarity with sequences of various phyllocoptines, KF782375 (*K. theae*, isolate 18sr6 18S)—99.39% similarity with sequence MZ279804 of *Fujianacarus wisterianis* (Eriophyidae: Phyllocoptinae: Phyllocoptini), and KF782586 (*K. theae*, isolate 28sd9-10r6 28S)—99.61% similarity with sequence MZ326423 of *F. wisterianis* and 80%–90% with sequences of some other phyllocoptines. These findings are in accordance with the results of our molecular phylogenetic analyses (Figure 6) and indicate that the sequences KF782375, KF782475, and KF782586 [10] belong to a non-nothopodine species, probably *F. wisterianis*.

#### 3.2.3. Comparative Mitogenomics

**Figure 7:**
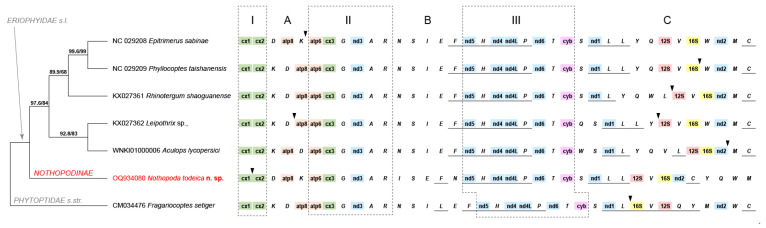
Maximum-likelihood phylogeny (12 mitochondrial protein genes, *12S,* and *16S*) of Eriophyoidea (**left**) and gene orders in the mitochondrial genomes included in the analysis (**right**). *Nothopoda todeica* **n. sp.** is colorized red. Branch labels are: SH-aLRT support (%)/ultrafast bootstrap support (UFBS, %). The constant blocks (I, II, and III) and variable zones (A, B, and C) of mitochondrial genes in seven eriophyoid mites species are indicated. Black arrowheads point to the position of control regions. Genes located on the negative chain of mitochondrial DNA are underlined. Notations: cx: cytochrome-c oxidase (green), atp: ATP synthase (orange), nd: NADH dehydrogenase (blue), cyb: cytochrome b (purple), *12S* and *16S*: rRNA genes (red and yellow). Two large eriophyoid lineages are indicated with gray italic font.

The mitochondrion genome of *Nothopoda todeica* **n. sp.** is 14018 bp long and includes 13 protein-coding genes, 22 tRNA genes, 2 rRNA genes, and 1 control region (Figure 7). Ten genes are located on the negative chain; four of them are protein-coding (*NAD1*, *NAD4*, *NAD4L*, and *NAD5*), and six genes code tRNAs (*F*, *H*, *P*, *L1*, *L2*, and *C*). The GC content is 16%. All protein-coding genes are terminated with the stop-codon TAA, except *NAD4* (TAG). The control region (*CR*) is located between genes *Cox1* and *Cox2*. In comparison to the other six eriophyoid species investigated to date [8,12,20,21,22], *CR* in *N. todeica* **n. sp.** Is, on average, six times longer (651 bp vs. 114 bp) and contains poly-AT repeats. 

All eriophyoids in our analyses possessed three stable blocks of mitochondrial genes: I (*Cox1*-*Cox2*), II (*ATP6*-*Cox3*-*G*-*NAD3*-*A*-*R*), and III (*NAD5*-*H*-*NAD4*-*NAD4L*-*P*-*NAD6*-*T*-*CYB*, genes located on the negative chain of mitochondrial DNA are underlined). Blocks I, II, and III were separated by three variable zones: A, B, and C (Figure 7). The short zone, A, contained differently ordered genes: *K*, *D*, and *ATP8*. The relatively constant zone B contained 5 or 6 tRNA genes, and the highly variable zone C contained 2 protein genes (*NAD1* and *NAD2*), 8 or 9 tRNA genes, and 2 rRNA genes (*12S* and *16S*). Contrary to all members of Eriophyidae s.l., in *Fragariocoptes setiger* (Phytoptidae s.str.), rRNA genes were situated on the negative chain of the mitochondrial DNA, corresponding to the position of these genes in a hypothetical ancestral mitogenome of Acariformes [8]. 

The main differences in the mitochondrial gene order between *N. todeica* **n. sp.** and other investigated eriophyoids were in the relative position of the tRNA genes in zones A, B, and C. *Nothopoda todeica* **n. sp.** has two unique gene clusters in zones B and C (cluster *I-S-E-F-N* prior to the gene *NAD5* and cluster *C-Y-Q-W-M* after the gene *NAD2*) and shares the same gene order in zone A (*D*-ATP8-*K*) with *Epitrimerus sabinae*.

The molecular phylogenetic analysis of the mitogenomic dataset revealed a tree topology of Eriophyoidea similar to that obtained in the analysis of the sequences of nuclear rRNA genes reported above (Figure 6 and Figure 7) and inferred Nothopodinae as the sister to all other members of Eriophyidae s.l. (with respect to phytoptids).

## 4. Discussion

In this study, we focused on a comprehensive investigation of the morphology and molecular phylogeny of *N. todeica* **n. sp.**, a new species of the subfamily Nothopodinae associated with a disjunct Afro-Australasian plant taxon, the fern *Todea barbara,* collected in South Africa. Morphologically, *N. todeica* **n. sp.** appeared to be a typical representative of *Nothopoda* that is very similar to some other members of this genus reported from eudicot trees from Asia, especially *N. camelliae.* Remarkably, both species share an interesting structure, a small subrhomboidal plate preceding the anal opening ([53], Figure 3B; this paper Figure 3H and Figure 4I). In *N. todeica* **n. sp.**, this plate is distinctly dotted, presumably porous, and may be associated with recently discovered elements of the anal secretory apparatus that serves for the excretion of anal gland secretions in Eriophyoidea [51]. 

The molecular phylogenetic analyses in this work were based on the dataset containing the sequences of nothopodines collected in China [10,12]. Similar to our previous work, when we used sequences from GenBank [15], we again found new erroneous sequences (KF782375, KF782475, and KF782586) wrongly assigned to the nothopodine genus *Kuangella*, instead of Phyllocoptinae. This emphasized the need to perform careful Blast verifications of the sequences before including them in the analyses and submitting them to publicly available databases, as well as the need to retest the hypotheses, investigated with erroneous sequences included.

In this work, we applied a very conservative approach for aligning sequences of nuclear rDNA genes, and, following [9,35], we removed all ambiguously aligned nucleotide positions corresponding to the hypervariable regions of *18S* and *28S* genes in order to avoid false nucleotide homologies in the alignments. Our analyses confirmed the basal position of Nothopodinae in Eriophyoidea s.l. They also rejected the monophyly of the tribes Colopodacini and Nothopodini, which was not surprising considering that the presence of coxal setae *1b* (defining Colopodacini) is a plesiomorphy, and the loss of this setae (defining Nothopodini) could have occurred homoplastically in different nothopodine taxa. Our results also show the nested position of the African fern-associated *Nothopoda* within a clade dominated by Asian nothopodines from angiosperms, which implies: (a) a likely secondary association of nothopodines with ferns and (b) no relation between geography (continents) and the phylogenetic relationships of Nothopodinae species. Considering the high concentration of nothopodine taxa in Asia and the results of our study, we hypothesized that Nothopodinae originated and diversified in Asia and, later (probably quite recently), dispersed to other continents. This hypothesis may be tested in the future, when more sequences of various nothopodine genera associated with larger sets of host plant orders are available for analyses.

Finally, in this study, we obtained a first complete annotated mitochondrial genome for Nothopodinae and found two unique clusters of tRNA genes adjacent to the protein-coding genes *NAD5* and *NAD2* in the mitogenome of *N. todeica* **n. sp.** Our mitogenomic analysis showed the separation of Nothopodinae from other members of Eriophyidae s.l., which corresponds with a notably deviating gene order in the mitogenome of *N. todeica* **n. sp.** and the basal position of Nothopodinae in Eriophyidae s.l. revealed in the analyses of *Cox1*, *18S*, and *28S* sequences. The question of whether the new gene order in the mitogenome of *N. todeica* **n. sp.** is a synapomorphy of the whole subfamily Nothopodinae or only an autapomophy of the new species needs further investigations. The results of the relatively recent pioneer works by Xue et al. [12,20] on the comparative mitogenomics of eriophyoids show the high uniformity of the mitochondrial gene order in Eriophyoidea. However, as novel annotated mitogenomes become available ([8,21,22] this paper), this view is gradually changing to the opposite, which implies high potential diversity and plasticity in the arrangement of mitochondrial genes in different lineages of gall mites. Continued research in this direction will contribute to resolving the phylogeny of Eriophyoidea.

## Figures and Tables

**Table 1:** Morphological differences between *Nothopoda todeica* **n. sp.** and *N. camelliae* Lv et al., 2022.

Character	*N. todeica* n. sp.	*N. camelliae*
Shape of microtubercles on coxal plates	Spine-shaped	Subspherical
Microtubercles on dorsal opisthosomal annuli	Absent	First five dorsal annuli with microtubercles, all other annuli with microtubercles present only midway
Microtubercles on ventral opisthosomal annuli	All ventral annuli completely microtuberculated	Microtubercules form three longitudinal bands separated by smooth areas
Microgranulations on femora I and II	Present only distally on ventral femora	Present on entire femora surface
Short thin lines within cells between ridges of prodorsal shield	Absent	Distinct, numerous
Host plant	*Todea barbara* (L.) T. Moore (Osmundaceae)	*Camellia oleifera* Abel (Theaceae)
Relation to host	Vagrant on lower surface of fronds	Vagrant on lower leaf surface
Reference	This paper	[53]

## Data Availability

All new DNA sequences obtained in this study have been deposited in the National Center for Biotechnology Information (NCBI) GenBank database (https://www.ncbi.nlm.nih.gov/genbank) (accessed on 3 May 2023).

## References

[1] Nuzzaci G., Alberti G., Lindquist E.E., Sabelis M.W., Bruin J. (1996). Internal anatomy and physiology. Eriophyoid Mites: Their Biology, Natural Enemies and Control.

[2] Sidorchuk E.A., Schmidt A.R., Ragazzi E., Roghi G., Lindquist E.E. (2015). Plant-feeding mite diversity in Triassic amber (Acari: Tetrapodili). J. Syst. Palaeontol..

[3] Propistsova E.A., Makarova A.A., Chetverikov P.E., Polilov A.A. (2023). Anatomy of the miniature four-legged mite *Achaetocoptes quercifolii* (Arachnida: Acariformes: Eriophyoidea). Arthropod Struct. Dev..

[4] Lindquist E.E., Lindquist E.E., Sabelis M.W., Bruin J. (1996). External anatomy and notation of structures. Eriophyoid Mites: Their Biology, Natural Enemies and Control.

[5] Bolton S.J., Chetverikov P.E., Klompen H. (2017). Morphological support for a clade comprising two vermiform mite lineages: Eriophyoidea (Acariformes) and Nematalycidae (Acariformes). Syst. Appl. Acarol..

[6] Bolton S.J., Bauchan G.R., Chetverikov P.E., Ochoa R., Klompen H. (2018). A rudimentary sheath for the smallest of “biting” chelicerae: The mouthparts of *Cunliffea* (Nematalycidae) and a new hypothesis on the origin of the stylet sheath of Eriophyoidea (Acariformes). Int. J. Acarol..

[7] Klimov P.B., OConnor B.M., Chetverikov P.E., Bolton S.J., Pepato A.R., Mortazavi A.L., Tolstikov A.V., Bauchan G.R., Ochoa R. (2018). Comprehensive phylogeny of acariform mites (Acariformes) provides insights on the origin of the four-legged mites (Eriophyoidea), a long branch. Mol. Phylogenet. Evol..

[8] Klimov P.B., Chetverikov P.E., Dodueva I.E., Vishnyakov A.E., Bolton S.J., Paponova S.S., Lutova L.A., Tolstikov A.V. (2022). Symbiotic bacteria of the gall-inducing mite *Fragariocoptes setiger* (Eriophyoidea) and phylogenomic resolution of the eriophyoid position among Acari. Sci. Rep..

[9] Pepato A.R., Costa S.G.D.S., Harvey M.S., Klimov P.B. (2022). One-way ticket to the blue: A large-scale, dated phylogeny revealed asymmetric land-to-water transitions in acariform mites (Acari: Acariformes). Mol. Phylogenet. Evol..

[10] Li H.S., Xue X.F., Hong X.Y. (2014). Homoplastic evolution and host association of Eriophyoidea (Acari, Prostigmata) conflict with the morphological-based taxonomic system. Mol. Phylogenet. Evol..

[11] Chetverikov P.E., Cvrković T., Makunin A., Sukhareva S., Vidović B., Petanović R. (2015). Basal divergence of Eriophyoidea (Acariformes, Eupodina) inferred from combined partial COI and 28S gene sequences and CLSM genital anatomy. Exp. Appl. Acarol..

[12] Xue X.F., Dong Y., Deng W., Hong X.Y., Shao R. (2017). The phylogenetic position of eriophyoid mites (superfamily Eriophyoidea) in Acariformes inferred from the sequences of mitochondrial genomes and nuclear small subunit (18S) rRNA gene. Mol. Phylogenet. Evol..

[13] Szudarek-Trepto N., Kaźmierski A., Skoracka A., Lewandowski M., Dabert J. (2022). Molecular Phylogeny Supports the Monophyly of the Mite Supercohort Eupodides (Acariformes: Trombidiformes) and Greatly Coincides with Traditional Morphological Definition of the Taxon. Ann. Zool..

[14] Amrine J.W., Stasny T.A.H., Flechtmann C.H.W. (2003). Revised Keys to the World Genera of the Eriophyoidea (Acari: Prostigmata).

[15] Chetverikov P.E., Bertone M. (2022). First rhyncaphytoptine mite (Eriophyoidea, Diptilomiopidae) parasitizing american hazelnut (*Corylus americana*): Molecular identification, confocal microscopy, and phylogenetic position. Exp. Appl. Acarol..

[16] Bolton S.J., Chetverikov P.E., Ochoa R., Klimov P.B. (2023). Where Eriophyoidea (Acariformes) belong in the tree of life. Insects.

[17] Shevchenko V.G., Bagnyuk I.G., Sukhareva S.I. (1991). A new family of Pentasetacidae (Acariformes, Tetrapodili) and its role in treatment of the origin and evolution of the group. Zool. Ž..

[18] Chetverikov P.E., Petanović R.U. (2016). Description of a new early-derivative mite, *Pentasetacus plicatus* n. sp. (Acariformes, Eriophyoidea), and remarks on the systematic position of pentasetacines. Zootaxa.

[19] Chetverikov P.E., Rector B.G., Tonkel K., Dimitri L., Cheglakov D.S., Romanovich A.E., Amrine J. (2022). Phylogenetic position of a new *Trisetacus* mite species (Nalepellidae) destroying seeds of North American junipers and new hypotheses on basal divergence of Eriophyoidea. Insects.

[20] Xue X.F., Guo J.F., Dong Y., Hong X.Y., Shao R. (2016). Mitochondrial genome evolution and tRNA truncation in Acariformes mites: New evidence from eriophyoid mites. Sci. Rep..

[21] Greenhalgh R., Dermauw W., Glas J.J., Rombauts S., Wybouw N., Thomas J., Alba J.M., Pritham E.J., Legarrea S., Feyereisen R. (2020). Genome streamlining in a minute herbivore that manipulates its host plant. eLife.

[22] Yin Y., Yao L.F., Zhang Q., Hebert P.D., Xue X.F. (2020). Using multiple lines of evidence to delimit protogynes and deutogynes of four-legged mites: A case study on *Epitrimerus sabinae* s.l. (Acari: Eriophyidae). Invertebr. Syst..

[23] Chase M.W., Christenhusz M.J.M., Fay M.F., Byng J.W., Judd W.S., Soltis D.E., Mabberley D.J., Sennikov A.N., Soltis P.S., Stevens P.F. (2016). An update of the Angiosperm Phylogeny Group classification for the orders and families of flowering plants: APG IV. Bot. J. Linn. Soc..

[24] Skoracka A., Smith L., Oldfield G., Cristofaro M., Amrine J.W. (2010). Host-plant specificity and specialization in eriophyoid mites and their importance for the use of eriophyoid mites as biocontrol agents of weeds. Exp. Appl. Acarol..

[25] Ferreira B.G., Álvarez R., Bragança G.P., Alvarenga D.R., Pérez-Hidalgo N., Isaias R.M. (2019). Feeding and other gall facets: Patterns and determinants in gall structure. Bot. Rev..

[26] Westphal E., Shorthouse J., Rohfritsch O. (1992). Cecidogenesis and resistance phenomena inmite-induced galls. Biology of Insect-Induced Galls.

[27] Parris B.S. (2001). Circum-Antarctic continental distribution patterns in pteridophyte species. Brittonia.

[28] Bomfleur B., Escapa I. (2019). A silicified *Todea* trunk (Osmundaceae) from the Eocene of Patagonia. PalZ.

[29] Carvalho M.R., Wilf P., Hermsen E.J., Gandolfo M.A., Cúneo N.R., Johnson K.R. (2013). First record of *Todea* (Osmundaceae) in South America, from the early Eocene paleorainforests of Laguna del Hunco (Patagonia, Argentina). Am. J. Bot..

[30] Bomfleur B., Grimm G.W., McLoughlin S. (2015). *Osmunda pulchella* sp. nov. from the Jurassic of Sweden—Reconciling molecular and fossil evidence in the phylogeny of modern royal ferns (Osmundaceae). BMC Evol. Biol..

[31] Amrine J.W., Manson D.C.M., Lindquist E.E., Sabelis M.W., Bruin J. (1996). Preparation, mounting and descriptive study of eriophyoid mites. Eriophyoid Mites: Their Biology, Natural Enemies and Control.

[32] Chetverikov P.E. (2016). Video projector: A digital replacement for camera lucida for drawing mites and other microscopic objects. Syst. Appl. Acarol..

[33] Katoh K., Misawa K., Kuma K., Miyata T. (2002). MAFFT: A novel method for rapid multiple sequence alignment based on fast Fourier transformation. Nucleic Acids Res..

[34] Katoh K., Rozewicki J., Yamada K.D. (2017). MAFFT online service: Multiple sequence alignment, interactive sequence choice and visualization. Brief. Bioinform..

[35] Pepato A.R., da Rocha C.E., Dunlop J.A. (2010). Phylogenetic position of the acariform mites: Sensitivity to homology assessment under total evidence. BMC Evol. Biol..

[36] Castresana J. (2000). Selection of conserved blocks from multiple alignments for their use in phylogenetic analysis. Mol. Biol. Evol..

[37] Talavera G., Castresana J. (2007). Improvement of phylogenies after removing divergent and ambiguously aligned blocks from protein sequence alignments. Syst. Biol..

[38] Dereeper A., Guignon V., Blanc G., Audic S., Buffet S., Chevenet F., Dufayard J.F., Guindon S., Lefort V., Lescot M. (2008). Phylogeny.fr: Robust phylogenetic analysis for the non-specialist. Nucleic Acids Res..

[39] Minh B.Q., Schmidt H.A., Chernomor O., Schrempf D., Woodhams M.D., von Haeseler A., Lanfear R. (2020). IQ-TREE 2: New Models and Efficient Methods for Phylogenetic Inference in the Genomic Era. Mol. Biol. Evol..

[40] Kalyaanamoorthy S., Minh B.Q., Wong T.K.F., von Haeseler A., Jermiin L.S. (2017). ModelFinder: Fast model selection for accurate phylogenetic estimates. Nat. Methods.

[41] FastQC: A Quality Control Tool for High throughput Sequence Data. https://www.bioinformatics.babraham.ac.uk/projects/fastqc/.

[42] Chen S., Huang T., Zhou Y., Han Y., Xu M., Gu J. (2017). AfterQC: Automatic filtering, trimming, error removing and quality control for fastq data. BMC Bioinform..

[43] Nurk S., Meleshko D., Korobeynikov A., Pevzner P.A. (2017). metaSPAdes: A new versatile metagenomic assembler. Genome Res..

[44] Jin J.-J., Yu W.-B., Yang J.-B., Song Y., dePamphilis C.W., Yi T.-S., Li D.-Z. (2020). GetOrganelle: A fast and versatile toolkit for accurate de novo assembly of organelle genomes. Genome Biol..

[45] Meng G., Li Y., Yang C., Liu S. (2019). MitoZ: A toolkit for animal mitochondrial genome assembly, annotation and visualization. Nucleic Acids Res..

[46] Okonechnikov K., Golosova O., Fursov M. (2012). Unipro UGENE: A unified bioinformatics toolkit. Bioinformatics.

[47] Seemann T. (2013). Barrnap 0.7: Rapid Ribosomal RNA Prediction. https://github.com/tseemann/barrnap.

[48] Quinlan A.R., Hall I.M. (2010). BEDTools: A flexible suite of utilities for comparing genomic features. Bioinformatics.

[49] Altschul S.F., Madden T.L., Schäffer A.A., Zhang J., Zhang Z., Miller W., Lipman D.J. (1997). Gapped BLAST and PSI-BLAST: A new generation of protein database search programs. Nucleic Acids Res..

[50] Bernt M., Donath A., Jühling F., Externbrink F., Florentz C., Fritzsch G., Pütz J., Middendorf M., Stadler P.F. (2013). MITOS: Improved de novo metazoan mitochondrial genome annotation. Mol. Phylogenet. Evol..

[51] Laslett D., Canbäck B. (2008). ARWEN, a program to detect tRNA genes in metazoan mitochondrial nucleotide sequences. Bioinformatics.

[52] Chetverikov P.E., Craemer C., Gankevich V.D., Vishnyakov A.E., Zhuk A.S. (2023). A New Webbing *Aberoptus* Species from South Africa Provides Insight in Silk Production in Gall Mites (Eriophyoidea). Diversity.

[53] Lv A., Deng X., Zhao Y., Du S., Tan M., Wang G. (2022). Three new species *Nothopoda* Keifer (Acari, Nothopodinae) from Guangxi, China. Int. J. Acarol..

[54] Keifer H.H. (1969). Eriophyoid Studies C-1.

[55] Meyer M.S., Ueckermann E.A. (1997). Afrotropical Eriophyoidea: On some species of the subfamily Nothopodinae (Acari: Eriophyidae). Acarologia.

[56] GBIF, Global Biodiversity Information Facility. https://www.gbif.org.

